# Mesh Fixation with Fibrin Sealant in Totally Extraperitoneal Hernia Repair

**DOI:** 10.1089/lap.2016.0555

**Published:** 2017-03-01

**Authors:** Hank Hirsch, Kei Nagatomo, Jonathan Gefen

**Affiliations:** Lankenau Medical Center, General Surgery, Wynnewood, Pennsylvania.

**Keywords:** totally extraperitoneal, TEP, fibrin sealant, inguinal hernia repair, mesh repair

## Abstract

***Introduction:*** Repair of inguinal hernia is a common procedure, but there is a lack of consensus as to the optimal repair technique along with the use of mesh and methods of mesh fixation. The objective of this study was to evaluate the efficacy and safety of fibrin sealant for mesh fixation in laparoscopic totally extraperitoneal (TEP) inguinal hernia repair.

***Materials and Methods:*** A study was conducted of the first 200 patients undergoing TEP hernia repair with mesh fixation using fibrin sealant between March 2012 and January 2014. The primary outcome measures were (1) chronic pain (persisting for >3 months), (2) persistence of hernia (recurrence identified within first 2 weeks postoperatively), (3) hernia recurrence, and (4) any additional perioperative complications. The mean follow-up in the series was 34.4 ± 6.1 months (range 22.2–44.1).

***Results:*** Of the 278 hernias repaired in 204 patients (74 bilateral, 130 unilateral), 38 were recurrent and 240 were primary. Three patients (1.5%) had a persistent hernia, including one with a planned return to the operating room the next day due to poor visualization. Three patients (1.5%) had a hernia recurrence. Twelve patients (5.9%) reported experiencing chronic pain. The remaining complications were minor and resolved over time.

***Conclusions:*** TEP repair of inguinal hernia using mesh secured with fibrin sealant can be effectively used to treat primary, recurrent, unilateral, and bilateral inguinal hernias in adults with minimal recurrence rates and complications during almost 3 years of follow-up.

## Introduction

Repair of inguinal hernia is a common procedure with over half a million performed per year in the United States.^1,2^ The primary goals of hernia repair include prevention of a potential strangulation, minimizing chance of recurrence, early return to normal activity and avoidance of acute and chronic pain. There is a lack of consensus as to the optimal repair technique, with controversy over the best approach, use of mesh, and methods of mesh fixation.

The laparoscopic transabdominal preperitoneal approach was introduced in the 1990s and refined to avoid entry into the abdominal cavity in the totally extraperitoneal (TEP) approach.^3,4^ TEP employs a balloon to dissect the preperitoneal space, which has helped the extraperitoneal approach advance as an expeditious and safe option for inguinal hernia repair.^5^

While laparoscopic hernia repair has become commonplace among surgeons with expertise in the technique,^6,7^ controversy remains over the ideal method of mesh fixation. The dominant techniques are tacks, fibrin sealant fixation, self-affixing mesh, or no fixation at all. A review of the literature found only one study conducted in the United States examining the use of fibrin sealant for mesh fixation,^8^ although a number of authors have described fibrin sealant use internationally.^9–17^ Mesh fixation remains an off-label indication for fibrin sealant in the United States, which may contribute to the paucity of data on its use.

The objective of this study was to evaluate the efficacy and safety of fibrin sealant (TISSEEL [Fibrin Sealant]; Baxter Healthcare Corp., Deerfield, IL) for mesh fixation in TEP hernia repair.

## Materials and Methods

A retrospective study was conducted of the first 200 patients undergoing TEP hernia repair with mesh fixation using fibrin sealant between March 2012 and January 2014 at a teaching community hospital. This data review was considered exempt under 45 CFR part 46 Category 4 as the existing data were recorded by the investigator in such a manner that subjects cannot be identified, directly or through identifiers linked to the subjects (“HIPAA Safe Harbor”). The primary outcome measures were (1) chronic pain (persisting for ≥3 months after the procedure), (2) persistence of hernia (defined as recurrence identified within the first 2 weeks postoperatively), (3) hernia recurrence, and (4) any additional perioperative complications. Demographic data and data related to morbidity and mortality were retrieved from a prospectively created database and retrospective review of the patients' records. All procedures were performed by a single surgeon. Continuous data are summarized as mean, standard deviation, and range unless otherwise stated. Categorical data are summarized using frequencies and percentages. All analyses were performed using SAS Version 9.3 (SAS Institute, Inc., Cary, NC).

### Surgical technique

A small, transverse incision is made within a skin fold of the umbilicus. The anterior rectus sheath ipsilateral to the hernia is incised vertically. The rectus muscle is retracted laterally, and a balloon dissector (Spacemaker Plus Balloon Dissector; Medtronic, Inc., Minneapolis, MN) is used to access the preperitoneal space. The space is insufflated to 12 mmHg. Two 5 mm working ports are placed through the contralateral rectus muscle below the level of the umbilicus. For bilateral hernia repairs, the ports are placed in the midline.

**FIG. 1.**
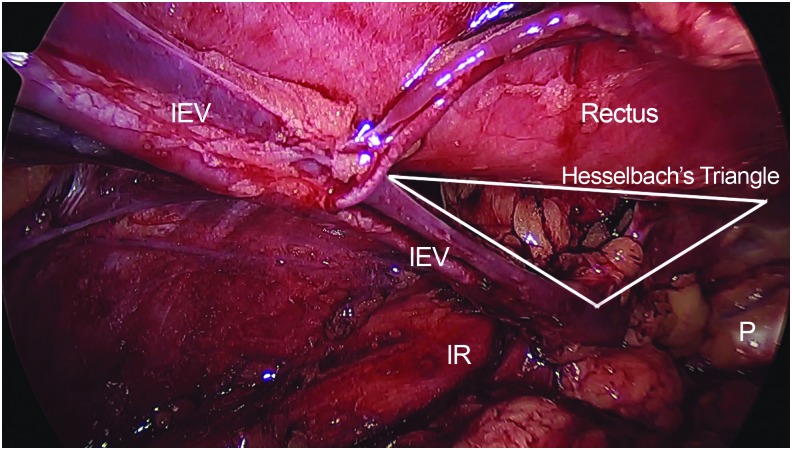
Hernia anatomy. IEV, inferior epigastric vessel; IR, inguinal ring; P, pubic bone.

Dissection proceeds from medial to lateral (Fig. 1). The hernia is bluntly reduced and the peritoneum is mobilized away from the abdominal wall. A 10 × 15 cm piece of mesh (ULTRAPRO Partially Absorbable Lightweight Mesh; Ethicon, Inc., Somerville, NJ) is inserted to cover the internal ring, Hesselbach's triangle, and the femoral canal with wide overlap in all directions.

**FIG. 2.**
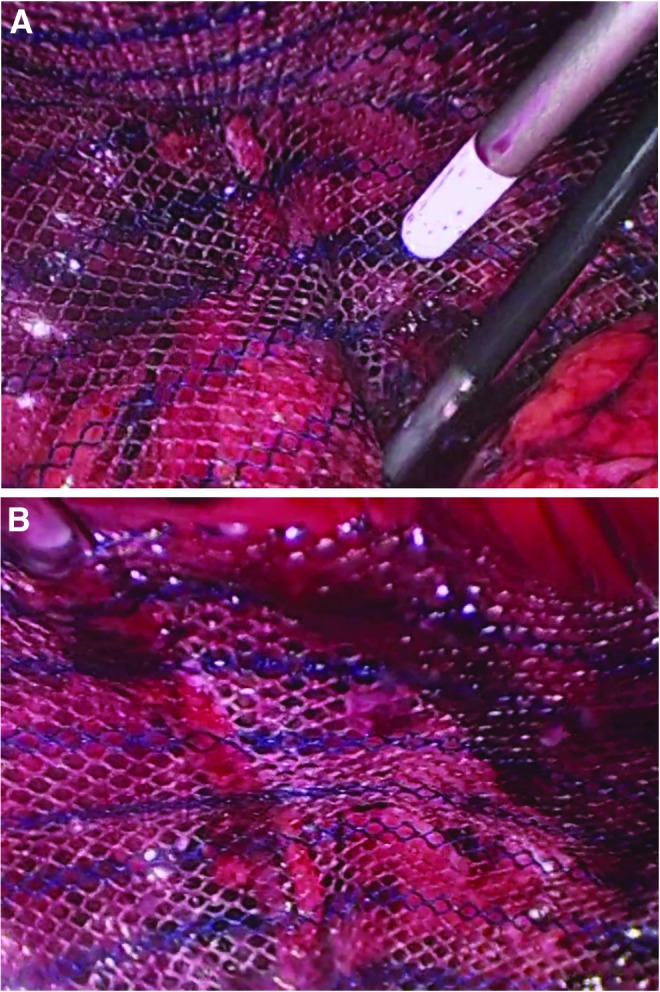
**(A)** Fibrin sealant (TISSEEL [Fibrin Sealant]; Baxter Healthcare Corp., Deerfield, IL.) application to mesh during TEP hernia repair. The applicator pictured (with white tip) was specifically designed for minimally invasive surgery. This new device allows for more efficient use and even distribution of fibrin sealant at lower volumes. An additional instrument (shown in lower right corner) holds the mesh in place during polymerization of the product (which occurs in ∼2 minutes). **(B)** Mesh in place following fixation with fibrin sealant. TEP, totally extraperitoneal.

Fibrin sealant (2–4 mL for unilateral repair, 8–10 mL for bilateral repair) is used for mesh fixation. A laparoscopic applicator is used to spray a thin layer of sealant on the mesh while a separate instrument holds the mesh in place during polymerization (∼2 minutes following application). The application device (DuploSpray MIS) allows for efficient and even distribution of fibrin sealant at low volumes (Fig. 2).

After final inspection, the space is desufflated under direct laparoscopic vision. The umbilical fascia is closed with a figure-of-eight 0 Vicryl suture. The skin is closed with 4-0 Monocryl subcuticular sutures.

## Results

From March 2012 to January, 2014, 204 patients (190 men, 14 women) underwent the TEP procedure with mesh fixation using fibrin sealant. The mean age was 55.3 ± 14.6 (range 22.0–90.0) and mean body mass index was 25.9 ± 3.2 (range 18.5–36.1). Comorbidities in the study population included heart disease (defined as coronary artery disease, arrhythmia, and valvular disease), lung disease (defined as asthma, obstructive sleep apnea, and chronic obstructive pulmonary disease), hypertension, diabetes, and prior abdominal surgery; Table 1.

**Table 1.** Baseline and Medical History Summary

	*Summary*
Age (years)	55.3 ± 14.6 (204) 55.0 [22.0, 90.0]
Gender	
Male	93.1% (190/204)
Female	6.9% (14/204)
BMI-all subjects	25.9 ± 3.2 (204) 25.8 [18.5, 36.1]
Heart disease	21.6% (44/204)
Lung disease	16.7% (34/204)
Hypertension	30.9% (63/204)
Diabetes	5.9% (12/204)

Data presented as sum, % (*n*/*N*), or mean ± SD (*N*); median [min, max].

BMI, body mass index; SD, standard deviation.

Of the 278 hernias repaired (74 bilateral, 130 unilateral), 38 were recurrent, and 240 were primary (Table 2). Including those with recurrent hernias, there were 55 patients who had undergone previous abdominal surgery. The majority of repairs were for indirect hernias (168/60%), followed by direct hernias (66/24%), and some with both direct and indirect components (44/15%). The mean procedure time for all subjects was 74 ± 24.5 minutes (range 33.0–210.0). Procedure time was further broken down into unilateral procedures that averaged 66.4 ± 23.5 minutes (range 33.0–210.0) and bilateral procedures that were 86.4 ± 21.0 minutes (range 50.0–179.0). The mean follow-up for all patients in the series was 34.4 ± 6.1 months (range 22.2–44.1). However, it is important to note that after the standard 8-week clinical visit, patients were contacted to assess their conditions, but not at prospectively determined regular intervals.

**Table 2.** Hernia and Procedural Summary

	*Summary*
Total number of hernias	278
Patients with primary hernia repair	81.4% (166/204)
Patients with recurrent hernias	18.6% (38/204)
Patients with prior abdominal surgery (includes recurrent hernias)	27.0% (55/204)
Hernia position	
Right	73.5% (150/204)
Left	62.7% (128/204)
Unilateral	63.7% (130/204)
Bilateral	36.3% (74/204)
Hernia type	
Indirect	60.4% (168/278)
Direct	23.7% (66/278)
Both (indirect/direct)	15.8% (44/278)
Procedure time (minutes)-all subjects	74.0 ± 24.5 (175) 71.0 [33.0, 210.0]

Data presented as sum, % (*n*/*N*), or mean ± SD (*N*); median [min, max].

SD, standard deviation.

### Complications

There were a total of 12 patients (5.9%) who reported experiencing chronic pain, defined as pain lasting over 3 months. In most cases, pain was mild and did not interfere with ADL's. One patient (0.5%) reported experiencing moderate pain at 1 year postoperatively. There were 3 patients (1.5%) with persistent hernia following repair, including one with poor laparoscopic visualization who returned to the operating room the next day for a planned, open repair. Three patients (1.5%) had a hernia recurrence in the study period (Table 3). There were 21 additional perioperative complications in 19 patients, including 2 patients that required immediate conversion to an open procedure (1 due to poor visualization, and 1 for an orchiopexy due to a retracted testicle that was found adherent to the hernia sac; Table 4). The remaining complications were minor and resolved over time and included dysuria, hyponatremia, seroma, urinary tract infection, urinary retention, and vomiting.

**Table 3.** Postoperative Complication Summary

*Complication type*	*Total events*	*Subjects with event*	*Percent of subjects*
Pain (lasting > 3 months)	12	12	5.9
Hernia persistence	3	3	1.5
Hernia recurrence	3	3	1.5
Total	18	18	8.8

**Table 4.** Perioperative Complication Summary

*Complication type*	*Total events*	*Subjects with event*	*Percent of subjects*
Conversion to open	2	2	1.0
Dysuria	1	1	0.5
Hyponatremia	1	1	0.5
Orchiopexy	1	1	0.5
Seroma	1	1	0.5
Urinary tract infection	1	1	0.5
Urinary retention	13	13	6.4
Vomiting	1	1	0.5
Total	21	19	9.3

## Discussion

This study collected data from the first 278 inguinal hernia repairs performed by a single surgeon utilizing a TEP approach with fibrin sealant for mesh fixation. The examination of results in a single surgeon series has the advantage of consistency in operative technique and postoperative patient management. The series was generated prospectively. However, the limits of this study include the inherent drawbacks of a retrospective review, and a lack of a control group and randomization.

While the optimal technique for inguinal hernia repair remains a topic of discussion among general surgeons, both open and minimally invasive approaches have proven to be safe and effective. In recent years, laparoscopic repair of an inguinal hernia has been shown to have several advantages over open repair including faster recovery and return to physical activities, decreased risk for acute and chronic postoperative pain, the ability to simultaneously repair bilateral hernias and, in the case of a recurrent hernia, the avoidance of reoperating on old scar tissue.^7,18–20^ The major drawbacks of laparoscopic approaches include the need for general anesthesia, longer operating times, and considerable expertise and experience required for the operating surgeon.^7,18–20^ Surgeon experience is also a key factor in the prevention of recurrence following laparoscopic hernia repair. Although a major early study indicated that recurrence was higher following laparoscopic procedures,^20^ more recent follow-up studies have shown that with increased surgeon experience, sufficient mesh coverage, and appropriate mesh fixation, recurrence rates are similar or lower following laparoscopic hernia repair.^18,19,21^

Although some early studies indicated that mesh fixation was unnecessary in TEP,^22,23^ secure fixation of mesh can help to reduce recurrence by preventing dislocation and improving the intended overlap of the hernia orifices.^10^ The use of fibrin sealant for fixation has been on the rise for hernia repairs of all types. It allows for avoidance of any sutures or tacks, which can potentially add to postoperative pain.^12,15,16^ With laparoscopic inguinal hernia repair, fibrin sealant has the added advantage of fixating the entire surface of the mesh. Fibrin sealant can be applied where tacks are prohibited, including the inferior and lateral portions of mesh overlying the external iliac vessels and sensory nerves (the “triangle of doom” and “triangle of pain” regions).^17^ A systematic review examining the use of fibrin sealant for mesh fixation in hernia repair showed distinct advantages over tissue-penetrating methods (staples, tacks) including a lower risk of postoperative complications and trend toward shorter recovery times, and lower rates of postoperative and chronic pain.^15^

As mentioned previously, although a number of international studies have been conducted to examine the use of fibrin sealant for mesh fixation in laparoscopic inguinal hernia repair,^9–17^ only one such study has previously been published in the United States.^8^ In his report in 2006, Fine studied 38 patients who had undergone TEP inguinal hernia repair with fibrin sealant for mesh fixation in 45 primary and 6 recurrent hernias. Only one hernia recurred (second recurrence of unilateral direct hernia) indicating a 2% hernia recurrence rate. Three patients (7.9%) reported chronic pain 12–18 months postoperatively. Although the incidence of pain was higher than that of our study (5.9%), it is still lower than the 9.2%–14.7% pain incidence that has been reported with the use of tacks^9,24^ and much less than up to 62% reported with open repair techniques.^25,26^

Tissue penetrating techniques of mesh fixation such as staples and tacks during laparoscopic inguinal repair is still a standard practice among surgeons, especially in the United States, despite its association with increased postoperative pain and the potential for vascular and nerve injuries.^11^ As demonstrated by the results of our study, and a number of international studies, fibrin sealant for mesh fixation in TEP repair is a reliable, safe, and effective option.

In conclusion, TEP repair of inguinal hernia using mesh secured with fibrin sealant can be effectively used to treat primary, recurrent, unilateral, and bilateral inguinal hernias in adults with minimal recurrence rates and complications as demonstrated by our positive outcomes in 278 hernia repairs during almost 3 years of follow-up.
